# Development and Validation of a Personalized, Sex-Specific Prediction Algorithm of Severe Atheromatosis in Middle-Aged Asymptomatic Individuals: The ILERVAS Study

**DOI:** 10.3389/fcvm.2022.895917

**Published:** 2022-07-14

**Authors:** Marcelino Bermúdez-López, Manuel Martí-Antonio, Eva Castro-Boqué, María del Mar Bretones, Cristina Farràs, Gerard Torres, Reinald Pamplona, Albert Lecube, Dídac Mauricio, José Manuel Valdivielso, Elvira Fernández

**Affiliations:** ^1^Grupo de Investigación Translacional Vascular y Renal, IRBLleida, Red de Investigación Renal (RedInRen-ISCIII), Lleida, Spain; ^2^Centre d’Atenció Primària Cappont, Gerència Territorial de Lleida, Institut Català de la Salut, Barcelona, Spain; ^3^Research Support Unit Lleida, Fundació Institut Universitari per a la Recerca a l’Atenció Primària de Salut Jordi Gol i Gorina (IDIAPJGol), Barcelona, Spain; ^4^Departament de Medicina Respiratòria, Hospital Universitari Arnau de Vilanova, Grup Recerca Translational Medicina Respiratòria, IRBLleida, Universitat de Lleida, Lleida, Spain; ^5^CIBER de Enfermedades Respiratorias (CIBERES), Madrid, Spain; ^6^Departament de Medicina Experimental, IRBLleida, Universitat de Lleida, Lleida, Spain; ^7^Departament d’Endocrinologia i Nutrició, Hospital Universitari Arnau de Vilanova, Grup de Recerca Obesitat i Metabolisme (ODIM), IRBLleida, Universitat de Lleida, Lleida, Spain; ^8^Centro de Investigación Biomédica en Red de Diabetes y Enfermedades Metabólicas Asociadas (CIBERDEM), Instituto de Salud Carlos III (ISCIII), Madrid, Spain; ^9^Departament d’Endocrinologia i Nutrició, Hospital de la Santa Creu i Sant Pau, Institut de Recerca Biomèdica Sant Pau (IIB Sant Pau), Barcelona, Spain

**Keywords:** cardiovascular disease, atherosclerosis, cardiovascular risk assessment, machine learning, recursive partitioning classification trees, vascular ultrasound

## Abstract

**Background:**

Although European guidelines recommend vascular ultrasound for the assessment of cardiovascular risk in low-to-moderate risk individuals, no algorithm properly identifies patients who could benefit from it. The aim of this study is to develop a sex-specific algorithm to identify those patients, especially women who are usually underdiagnosed.

**Methods:**

Clinical, anthropometrical, and biochemical data were combined with a 12-territory vascular ultrasound to predict severe atheromatosis (SA: ≥ 3 territories with plaque). A Personalized Algorithm for Severe Atheromatosis Prediction (PASAP-ILERVAS) was obtained by machine learning. Models were trained in the ILERVAS cohort (*n* = 8,330; 51% women) and validated in the control subpopulation of the NEFRONA cohort (*n* = 559; 47% women). Performance was compared to the Systematic COronary Risk Evaluation (SCORE) model.

**Results:**

The PASAP-ILERVAS is a sex-specific, easy-to-interpret predictive model that stratifies individuals according to their risk of SA in low, intermediate, or high risk. New clinical predictors beyond traditional factors were uncovered. In low- and high-risk (L&H-risk) men, the net reclassification index (NRI) was 0.044 (95% CI: 0.020–0.068), and the integrated discrimination index (IDI) was 0.038 (95% CI: 0.029–0.048) compared to the SCORE. In L&H-risk women, PASAP-ILERVAS showed a significant increase in the area under the curve (AUC, 0.074 (95% CI: 0.062–0.087), *p*-value: < 0.001), an NRI of 0.193 (95% CI: 0.162–0.224), and an IDI of 0.119 (95% CI: 0.109–0.129).

**Conclusion:**

The PASAP-ILERVAS improves SA prediction, especially in women. Thus, it could reduce the number of unnecessary complementary explorations selecting patients for a further imaging study within the intermediate risk group, increasing cost-effectiveness and optimizing health resources.

**Clinical Trial Registration:**

[www.ClinicalTrials.gov], identifier [NCT03228459].

## Introduction

Atherosclerotic cardiovascular disease (ASCVD) is still the main preventable cause of cardiovascular mortality and disability. ASCVD is a latent and progressive condition in which atheroma plaques develop in the artery wall, and it is usually widely extended by the time symptoms, typically a cardiovascular event, occur. Current European guidelines on cardiovascular prevention recommend initial assessment and stratification of the risk of ASCVD based on a probabilistic tool that includes traditional risk factors, followed by therapeutic intervention when necessary (1). However, these traditional risk scores underestimate individual risk in several circumstances, such as women, youths, and individuals with a low-to-moderate ASCVD risk (2, 3). Indeed, the number of cardiovascular events in individuals with a low-to-moderate risk is unacceptably high (4) and, although ASCVD is often thought to be a disease with a higher prevalence in men, the annual cardiovascular mortality rate has remained greater in women (5). Despite these numbers, women have been underrepresented in CVD-related clinical trials, and data are not often stratified by sex, limiting their interpretation. Indeed, better individualized prediction algorithms are urgently required, especially in individuals with moderate risk and in women.

One of the available tools to improve prediction algorithms is the use of non-invasive imaging techniques. Those techniques can detect the presence, estimate the extent, and evaluate the clinical consequences of ASCVD. Detection of subclinical atheromatosis in the arteries by vascular ultrasound is a reliable method to predict future coronary events (6), and it improves cardiovascular risk assessment in asymptomatic individuals (7). The most common method of estimating ASCVD burden has been carotid ultrasound. Carotid artery atheromatosis has a well-demonstrated role in the incidence of cerebrovascular and cardiovascular events (8). Atherosclerotic lower extremity peripheral artery disease is increasingly recognized as an important cause of cardiovascular morbidity and mortality (9). Nevertheless, femoral artery atheromatosis assessment has long been underexplored, although femoral atheroma plaques show the strongest association with cardiovascular risk factors, and a higher sensitivity to predict calcified coronary disease (10). Furthermore, extensive vascular territory assessment has been shown to increase its predictive value in cardiovascular mortality (11). However, and even though the 2019 European guideline recommends vascular ultrasounds in individuals with a low-to-moderate ASCVD risk (1), its implementation is still limited possibly because it is not easy to select candidates who could benefit from a comprehensive imaging study. Thus, a new algorithm that helps clinicians to identify those patients is highly recommended, as it will be cost-effective and will optimize health resources allocation.

Machine learning (ML) technologies can improve the diagnosis of diseases developing risk prediction algorithms to guide clinical decisions (12). Although several ML algorithms have been described to predict cardiovascular events (13–17), few predict the presence and extent of subclinical atheromatosis (18), and sex-specific algorithms are not available. Therefore, we developed and validated a personalized model to predict the probability of severe atheromatosis (SA; the PASAP-ILERVAS) based on classification trees (19), integrating clinical data, anthropometrical parameters, and routine, easy-to-obtain, affordable biochemical parameters in an asymptomatic, middle-aged population with a low-to-moderate cardiovascular risk. The new algorithm could help clinicians to select patients who could benefit from further vascular ultrasound examination and reduce the number of unnecessary complementary explorations.

## Materials and Methods

### Study Design and Selection of Participants

#### Discovery Cohort: The ILERVAS Study

It is an ongoing randomized, interventional, longitudinal clinical trial in a low-to-moderate cardiovascular risk population of the North-East region of Spain (ClinicalTrials.gov identifier: NCT03228459). The study was originally designed with two arms: (i) intervention group, thereafter called Mobile Unit follow-up group, and (ii) no intervention group, thereafter called Electronic Medical Record follow-up group. The intervention was the generation of a report sent to the primary care physician, including vascular ultrasound examination in carotid and femoral arteries assessing 12 territories, combined with clinical, anthropometric, lifestyle, and biochemical parameters. The study population was randomly allocated to the groups by stratified sampling from the electronic clinical history database of primary care. The no intervention group will be used to compare the impact of the intervention on cardiovascular morbidity and mortality. Thus, this group was not used for the present work. The intervention group was formed by 8,330 participants who were enrolled from January 2015 to December 2018 from 32 basic health areas of the province of Lleida (Catalonia, Spain). The study was designed to (i) assess the prevalence, vascular distribution, severity, and progression of subclinical atheromatosis in a middle-aged population with a low-to-moderate cardiovascular risk; (ii) uncover potential new factors predicting severe; and (iii) assess the impact of subclinical atheromatosis detection on the incidence of cardiovascular events during a 10-year follow-up period. The inclusion criteria were: 50–70-year-old women and 45–65-year-old men with at least one of the following CVD risk factors: hypertension, dyslipidemia, obesity (defined as body mass index, BMI ≥ 30), smoking, and/or first-degree relative who developed premature CVD (with a threshold at age 55 years for men or 65 years for women). The exclusion criteria were clinical history of diabetes, chronic kidney disease, cardiovascular pathology (angina, myocardial infarction, cerebral vascular accident, peripheral arterial disease, intestinal, or other ischemia), history of arterial surgery, active neoplasia, less than 18 months of expected life, long-term home care, and/or institutionalized population. The Ethics Committee of the Hospital Arnau de Vilanova (Lleida, Spain) approved the protocol (CEIC-1410). All patients signed informed consent. The study was conducted according to the principles of the Declaration of Helsinki. A more-detailed explanation of the study has been previously published (3).

#### Validation Cohort: The NEFRONA Study

It is an observational, multicenter, prospective study. For the present study, 559 healthy asymptomatic individuals with normal kidney function were selected from the NEFRONA cohort. Subjects were enrolled in Primary Care centers from October 2010 to June 2012. It was designed to evaluate the prevalence and natural history of subclinical atheromatosis in chronic kidney disease patients, and the contribution of vascular ultrasound for a more precise cardiovascular risk assessment. The NEFRONA cohort included a control group of individuals with normal kidney function, which is the subgroup used for the validation. Inclusion criteria were asymptomatic individuals between 18 and 74 years of age with a glomerular filtration rate (GFR) over 60 ml/min/1.73 m^2^. Exclusion criteria were active infections, pregnancy, active neoplasia, life expectancy shorter than 12 months, previous cardiovascular event, carotid surgery, or any organ transplantation. The Ethics Committee of the Hospital Arnau de Vilanova (Lleida, Spain) approved the protocol. All patients signed informed consent. The study was conducted according to the principles of the Declaration of Helsinki. A more-detailed explanation of the study has been previously published (20).

### Source of Information and Data Collection

Sociodemographic variables and clinical history of cardiovascular risk factors were collected from clinical records. In contrast, anthropometric data, smoking habit, and blood samples were collected at the moment of vascular examination.

### Anthropometric Data

The same protocols were used in both cohorts. Weight and height were measured according to guidelines to obtain BMI. Waist perimeter was measured with a non-stretchable tape with a precision of 0.1 cm to assess abdominal adiposity, which was defined as a waist perimeter ≥ 88 cm in women and ≥ 102 cm in men. Blood pressure was determined in triplicate, after 5 min rest using an automated device (Omron M6 Comfort, Omron Healthcare, Japan) at 2-min intervals. The mean of the three recordings was calculated.

### Biochemical Parameters

In the ILERVAS cohort, a fasting dried blood spot sample was obtained by a fingertip puncture according to standard protocols. Creatinine, uric acid, and total cholesterol levels were assessed with the REFLOTRON^®^ Plus system (Roche Diagnostics, Germany). It is a validated clinical chemistry system with highly correlated results to well-standardized laboratory methods (21–23). The glycosylated hemoglobin test was performed using a point-of-care instrument (Cobas B101^®^, Roche Diagnostics, Germany) that meets the generally accepted performance criteria for its measurement (24). In the NEFRONA cohort, biochemical parameters were obtained from a routine fasting blood test taken no more than 3 months apart from vascular examination. GFR was estimated according to international guidelines using the CKD-EPI equation in both cohorts (25).

### Atheromatous Plaque Assessment by Vascular Ultrasound

Vascular ultrasound was performed by nurses specialized in vascular imaging. Standardized scanning and reading protocols were followed to decrease interoperator variability and type 2 errors. Intra-observer reliability assessment showed a k-coefficient of one (two repeated measurements; 1,007 observations). Overall inter-rater reliability for all operators showed a k-coefficient of 0.915 (95% CI: 0.892–0.944; 959 observations). Readers were unaware of the patients’ clinical histories. The VIVID i BT09 model ultrasound system (GE Healthcare) equipped with a 12L-RS linear probe (6–13 MHz), and a pulsed Doppler ultrasound was used to assess hemodynamic repercussions. In the ILERVAS cohort, arterial ultrasound was performed in 12 territories, both carotid (common, bifurcation, internal, and external) and femoral (common and superficial) arteries. In the NEFRONA study, 10 territories were explored, both carotid (common, bifurcation, and internal) and femoral (common and superficial) arteries (3, 20). Subclinical atheromatosis was defined as the presence of any plaque in the explored areas. According to Mannheim consensus, an atheroma plaque was defined as a focal encroachment into the lumen of the artery ≥ 1.5 mm (26). SA was defined as ≥ 3 territories with atheroma plaque.

### Statistical Analysis

#### Variable Selection

Parameters that require a high degree of specialization or technical resources were ruled out. Thus, a set of easy-to-obtain and affordable variables were selected. The outcome was SA, defined as ≥ 3 territories with atheroma plaque out of 12. The explanatory variables were age, sex, clinical data (history of hypertension and dyslipidemia and smoking habit), anthropometrical data [systolic blood pressure (SBP), diastolic blood pressure (DBP), body mass index (BMI), abdominal adiposity, and waist-to-height ratio], and biochemical parameters (creatinine, GFR, uric acid, and total cholesterol).

#### Descriptive Analysis of the Cohorts

The clinical characteristics of the ILERVAS cohort (discovery cohort) and NEFRONA cohort (external validation cohort) were described as frequencies for categorical variables, and means and standard deviation (*SD*) for quantitative variables. Differences between SA and non-SA were assessed by several univariate logistic regressions for each clinical predictor adjusted by age and stratified by sex. The *p*-value of the likelihood-ratio test and odds ratio (OR) values with 95% confidence intervals (95% CI) were represented with the forestplot package (27). For each numerical predictor, adjusted OR per 1-*SD* higher parameter measure was estimated.

#### Sample Balancing

The descriptive analyzed revealed that the prevalence of SA was lower than non-SA in the discovery cohort. Thus, a Random Over-Sampling Example (ROSE) function (28) was used to increase the sample size of individuals with SA separately for men and women. Individuals who presented missing data (Supplementary Table 1) were excluded prior to sample balancing. This new balanced data set was used to train ML models in both sexes.

#### Machine Learning

A Recursive PARTicioning (rpart) classification tree (29) approach was used and plotted with rpart.plot (30). Trees were constructed in the balanced sample stratified by sex as follows: first, the algorithm recursively selected itself the clinical predictors that provide an optimal split in each node based on the generalized Gini index to obtain the maximal impurity reduction. The growth of the tree was controlled by pre-pruning techniques. A minimum of 200 individuals in each node was required to try a split and at least 100 in the terminal nodes. The maximum depth of the final tree was fixed on 6. Second, the resultant tree was trimmed back to minimize overfitting by post-pruning techniques, which select the complexity parameter (CP) that produced the minimum internal three-fold cross-validation error to prune the full tree. The probability of SA and the 95% CI were represented in the terminal nodes with ggplot2 (31). The obtained model was called Personalized Algorithm for Severe Atheromatosis Prediction (PASAP-ILERVAS).

#### Variable Importance Calculation

A variable importance score was calculated using the improvement measure attributable to each predictor in its role as splitter, plus the goodness for all splits in which it was a surrogate. The values of importance were scaled up to sum 100%.

#### Probability Calibration

In order to agglutinate terminal nodes into groups with similar risk, the probabilities of SA were calibrated using a histogram-transformed method using the CalibratR (32). First, the original probabilities with a maximum of 10 bins were plotted. Then, the optimal number of partitions to maximize the sensitivity was tested. The calibration process evidenced three risk groups, classified as low-, intermediate-, and high-risk nodes.

#### Prediction of Severe Atheromatosis and Non-severe Atheromatosis With Personalized Algorithm for Severe Atheromatosis Prediction

The Youden index (33) was computed to identify the cut-off probability to distinguish between non-SA and SA in the terminal nodes. Terminal nodes with a lower or equal probability to the cut-off were considered as non-SA, whereas terminal nodes with a higher probability were classified as SA.

#### Performance Metrics

The area under the curve (AUC) and the recommended metrics derived from the confusion matrix were used to evaluate model performance (34). A report from the European Society of Cardiology Prevention of CVD Programme is recommended to familiarize the reader with the risk prediction tools in cardiovascular disease prevention and performance metrics (35).

#### External Validation

The PASAP-ILERVAS model was externally validated in a subpopulation of the NEFRONA study and performance metrics were also calculated.

#### Systematic COronary Risk Evaluation Model

A logistic binary model with the Systematic COronary Risk Evaluation (SCORE) model was developed in the discovery balanced sample. The Youden index was computed to identify the cut-off probability to distinguish between non-SA and SA and performance metrics were calculated in L&H-risk individuals.

#### Improvement of Risk Prediction Between the Personalized Algorithm for Severe Atheromatosis Prediction and the Systematic COronary Risk Evaluation Model

The increment on AUC, the Net Reclassification Index (NRI), and the Integrated Discrimination Index (IDI) were evaluated to quantify the differences between both models in L&H-risk individuals (36). The SCORE model was considered as reference, and the PASAP-ILERVAS as the new model whose improvement was evaluated (37, 38). The CI for all metrics was performed by bootstrapping (2,000 bootstraps). The comparisons between models were performed by the DeLong test at 95% CI (39). To test the differences between performance metrics [sensitivity, specificity, accuracy, and the positive predictive value (PPV)], a two-sample test for equality of proportions with continuity correction was performed (40).

## Results

### Clinical Characteristics of the Discovery and Validation Cohorts

**FIGURE 1:**
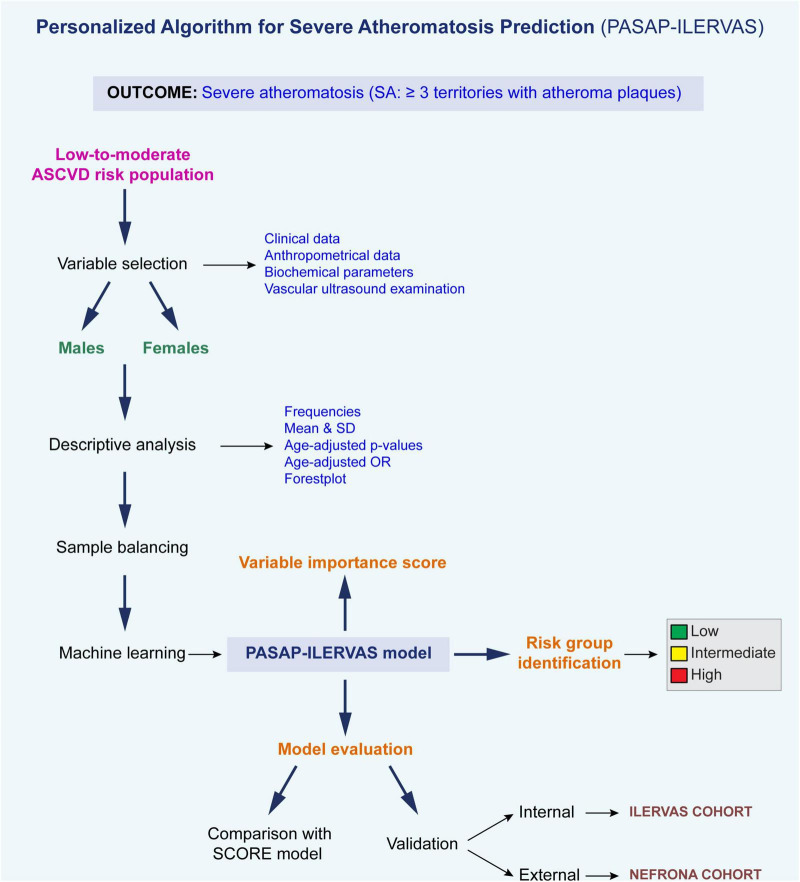
Analysis pipeline. ASCVD, atherosclerotic cardiovascular disease; OR, odds ratio; *SD*, standard deviation.

The workflow of the whole analysis is shown in Figure 1. The clinical characteristics of the ILERVAS cohort stratified by sex is shown in Table 1. The prevalence of SA, defined as ≥ 3 territories with atheroma plaque out of the 12 studied, was 42.6 and 24.4% in men and women, respectively. These patients showed a higher prevalence of clinical history of hypertension and dyslipidemia, and there were fewer non-smokers and more current smokers. The mean age was higher in participants with SA, and the levels of systolic blood pressure, glycosylated hemoglobin, uric acid, and total cholesterol. However, the association of these clinical predictors with SA showed some differences between sexes (Supplementary Figure 1). Age, systolic and diastolic blood pressure, total cholesterol, and being underweight showed a stronger positive association with SA in men than in women (OR: 1.76 vs. 1.51; OR: 1.32 vs. 1.23; OR: 1.17 vs. 1.07; OR: 1.24 vs. 1.13; and OR: 7.02 vs. 3.39, respectively). On the contrary, clinical history of hypertension showed a stronger positive association with SA in women than in men (OR: 1.58 vs. 1.24, respectively). Finally, the levels of glycosylated hemoglobin, uric acid, and clinical history of dyslipidemia showed a similar positive association in both sexes (OR: 1.11 vs. 1.09; OR: 1.10 vs. 1.10; and OR: 1.19 vs. 1.21 in men vs. women, respectively; Supplementary Table 2).

**TABLE 1:** Baseline characteristics of the ILERVAS cohort by sex.

	Men			Women		
	*n* = 4,108			*n* = 4,222		
		
	Severe atheromatosis			Severe atheromatosis		
			
	No	Yes	*P*-value	No	Yes	*P*-value
*n* (%)	2,358 (57.4%)	1,750 (42.6%)		3,190 (75.6%)	1,032 (24.4%)	
Age, years	53.68 (5.77)	56.87 (5.56)	<0.001	59.17 (5.83)	61.59 (5.99)	<0.001
**Clinical history**, *n* (%)						
Hypertension	832 (35.3%)	797 (45.5%)	0.002	1,205 (37.8%)	549 (53.2%)	<0.001
Dyslipidemia	1,171 (49.7%)	959 (54.8%)	0.007	1,729 (54.2%)	638 (61.8%)	0.012
Smoking habit			<0.001			<0.001
Non-smoker	737 (31.3%)	252 (14.4%)		1,797 (56.3%)	502 (48.6%)	
Former	912 (38.7%)	703 (40.2%)		728 (22.8%)	203 (19.7%)	
Current	709 (30.1%)	795 (45.4%)		665 (20.8%)	327 (31.7%)	
**Anthropometrical data**						
SBP, mmHg	130.60 (15.25)	136.51 (16.51)	<0.001	127.98 (17.15)	133.29 (16.94)	<0.001
DBP, mmHg	83.79 (9.32)	85.34 (9.48)	<0.001	79.04 (9.02)	79.59 (8.93)	0.060
Body mass index, *n* (%)			0.098			0.028
Underweight	2 (0.1%)	9 (0.5%)		11 (0.3%)	11 (1.1%)	
Normal weight	396 (16.8%)	283 (16.2%)		792 (24.8%)	251 (24.3%)	
Overweight/obesity	1,960 (83.1%)	1,457 (83.3%)		2,386 (74.8%)	770 (74.6%)	
Abdominal adiposity, *n* (%)	1,158 (49.1%)	914 (52.3%)	0.688	2,713 (85.1%)	892 (86.4%)	0.847
Waist-to-height ratio	0.60 (0.07)	0.60 (0.07)	0.907	0.63 (0.08)	0.64 (0.08)	0.837
**Biochemical parameters**						
Creatinine, mg/dL	0.90 (0.21)	0.88 (0.18)	<0.001	0.71 (0.16)	0.71 (0.16)	0.181
GFR, mL/min/1.73 m^2^	93.76 (14.35)	93.01 (13.99)	0.094	90.17 (14.09)	88.79 (14.24)	0.007
Glycosylated hemoglobin, %	5.49 (0.44)	5.56 (0.46)	0.002	5.56 (0.38)	5.61 (0.39)	0.014
Uric acid, mg/dL	6.09 (1.36)	6.25 (1.45)	0.003	4.69 (1.25)	4.86 (1.38)	0.007
Total cholesterol, mg/dL	196.49 (36.02)	203.03 (39.88)	<0.001	211.39 (36.24)	214.98 (37.86)	<0.001

*Values are shown as means and standard deviations for quantitative variables. Clinical history data were obtained from electronic medical records and refers to patients who had the prior clinical diagnostic of hypertension or dyslipidemia. Underweight was defined as a BMI < 18.5 kg/m^2^, normal weight as 18.5–24.9, overweight 25–29.9, and obesity ≥ 30. Abdominal adiposity was defined as an abdominal perimeter ≥ 88 cm in women or ≥ 102 in men. DBP, diastolic blood pressure; GFR, glomerular filtration rate; SBP, systolic blood pressure.*

### A Personalized Algorithm for Severe Atheromatosis Prediction

**FIGURE 2:**
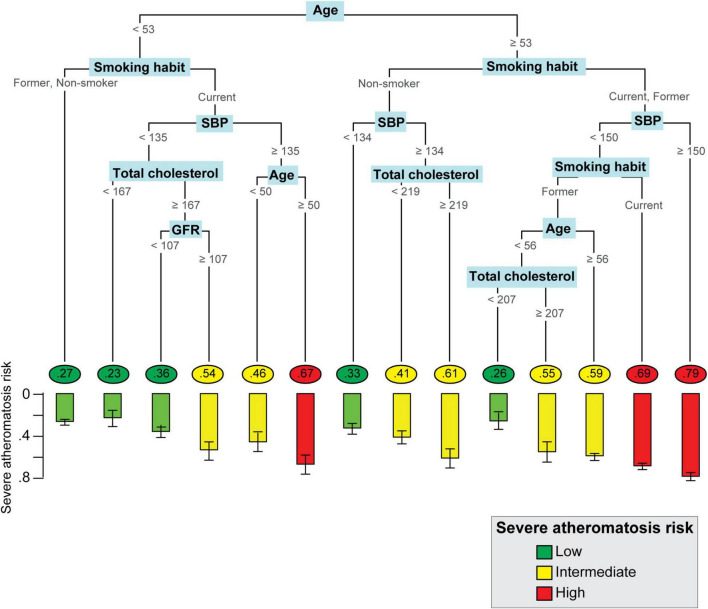
Personalized Algorithm for Severe Atheromatosis Prediction in men. The structure of the classification tree in men is represented. The probability of severe atheromatosis, which corresponds to the proportion of affected patients, is shown inside circles in the final nodes. The barplot offers a clearer visualization of this prevalence with its 95% confidence interval. The colors green, yellow, and red indicate the level of risk (low, intermediate, or high) identified after calibration. The units were as follows: age, year; GFR, ml/min/m^2^; SBP, mmHg; total cholesterol, mg/dl. GFR, glomerular filtration rate, SBP, systolic blood pressure.

**FIGURE 3:**
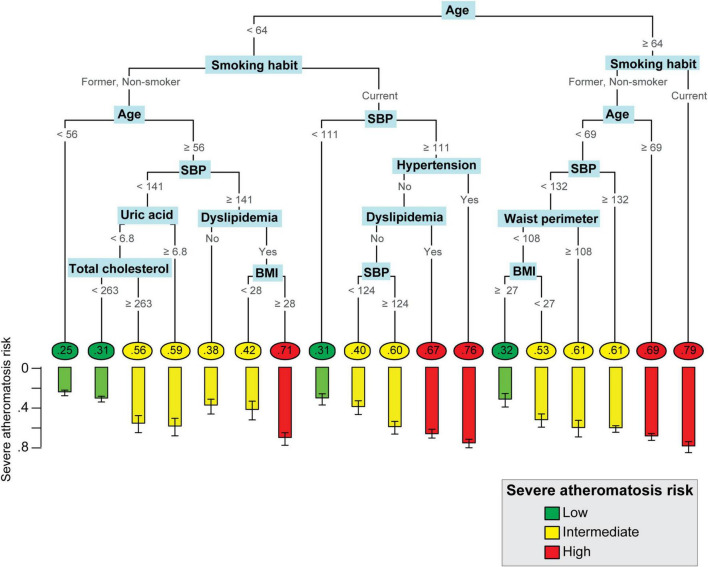
Personalized Algorithm for Severe Atheromatosis Prediction in women. The probability of severe atheromatosis, which corresponds to the proportion of affected patients, was shown inside circles in the final nodes. The barplot offers a clearer visualization of this prevalence with its 95% confidence interval. The colors green, yellow, and red indicate the level of risk (low, intermediate, or high, respectively) identified after calibration. Hypertension and dyslipidemia refer to patients who had prior clinical diagnostic of hypertension or dyslipidemia in their clinical records. The units were as follows: Age: year; BMI: kg/m^2^; SBP, mmHg; total cholesterol: mg/dL; uric acid: mg/dl; waist perimeter: cm. BMI, body mass index; SBP, systolic blood pressure.

Pruning the trees to 13 splits in men and 17 splits in women showed the lowest internal cross-validation error, beyond which tree complexity entailed no additional improvement (Supplementary Figure 2). The structure of the classification tree in men and women in the balanced ILERVAS cohort is shown in Figures 2, 3, respectively. In both genders, patients were first classified by age, followed by the smoking habit. The combination of these two parameters conditioned the clinical threshold of the other predictors, such as systolic blood pressure, total cholesterol, uric acid, GFR, BMI, waist perimeter, and history of dyslipidemia and hypertension.

A total number of 14 final nodes were obtained in men and 18 in women. The probability of severe atheromatosis and its 95% CI are shown in each node to improve visualization. In order to agglutinate individual terminal nodes into groups with similar risk, the probability of severe atheromatosis in each node was calibrated. Supplementary Figure 3 shows the absolute frequency of patients with or without severe atheromatosis and its probability of having the disease before and after calibration. Three risk profiles were identified: low-risk nodes with a mean prevalence of severe atheromatosis of 28.8% in men and 28.6% in women, intermediate-risk nodes with 54.8% in men and 54.9% in women, and high-risk nodes with 71.8% in men and 71.2% in women. These profiles are shown in Figures 2, 3 as green, yellow, and red nodes, respectively. Thus, clinicians could easily identify the risk of severe atheromatosis according to the characteristics of the patients.

**FIGURE 4:**
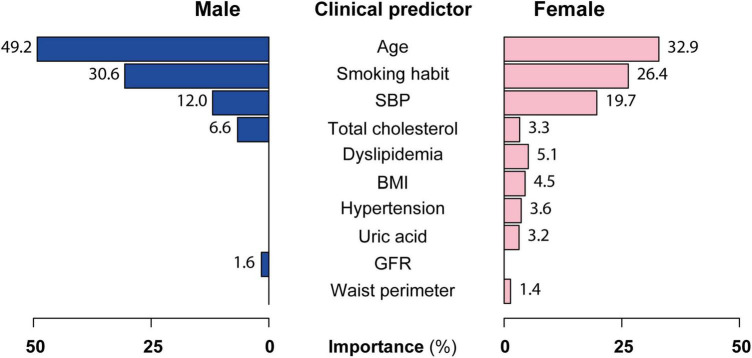
Clinical predictor importance in the PASAP-ILERVAS in both sexes. A variable importance score was calculated using the improvement measure attributable to each predictor in its role as splitter, plus the goodness for all splits in which it was a surrogate. The values of importance were scaled up to sum 100%. SBP, systolic blood pressure; BMI, body mass index; GFR, glomerular filtration rate.

Variable importance assessment revealed that in both sexes, age, smoking habit, SBP, and total cholesterol were the most important clinical predictors of severe atheromatosis, which account for 98.4% of the predictor importance in men and 82.3% in women. Additionally, a GFR ≥ 107 ml/min/m^2^ increased the risk in men. In women, other predictors, such as history of dyslipidemia, BMI, history of hypertension, uric acid, and waist perimeter were identified (Figure 4).

### Internal Validation of the Personalized Algorithm for Severe Atheromatosis Prediction Algorithm

Table 2 shows the internal validation metrics. Specificity, PPV, accuracy, and AUC were higher in L&H-risk individuals compared to the total sample (*p*-value: < 0.001), which indicate a higher feasibility of the PASAP-ILERVAS in those patients. On the contrary, the negative predictive value (NPV) showed no differences (*i*-value: > 0.1), and sensitivity was lower in L&H-risk individuals than in the total sample (*i*-value: < 0.001).

**TABLE 2:** Internal and external validation of the PASAP-ILERVAS.

	PASAP-ILERVAS			
	
	Internal validation		External validation	
		
	Men	Women	Men	Women
**Absence of severe atheromatosis**	0.500	0.499	0.688	0.893
Negative predictive value	0.688	0.695	0.876	0.940
Low and high risk	0.712	0.714	0.898	0.941
Specificity	0.628	0.614	0.624	0.710
Low and high risk	0.763	0.753	0.795	0.774
**Prevalence of severe atheromatosis**	0.500	0.501	0.312	0.107
Positive predictive value	0.658	0.656	0.493	0.205
Low and high risk	0.718	0.712	0.561	0.178
Sensitivity	0.715	0.733	0.805	0.625
Low and high risk	0.661	0.669	0.744	0.500
**Accuracy**	0.671	0.673	0.681	0.701
Low and high risk	0.715	0.713	0.782	0.750
**AUC**	0.710	0.708	0.755	0.693
Low and high risk	0.734	0.730	0.799	0.691

In L&H-risk patients, all parameters were similar in both sexes. The NPV, the PPV, and accuracy were 0.71. Thus, the PASAP-ILERVAS showed a 71% of correct predictions (including both true positive and true negative). Finally, AUC was 0.734 in men and 0.730 in women.

### External Validation of the Personalized Algorithm for Severe Atheromatosis Prediction Algorithm

The external validation was performed in an asymptomatic, kidney disease-free, subpopulation of the NEFRONA cohort. Although the prevalence of SA was lower in the NEFRONA cohort compared to the ILERVAS cohort, patients showed similar characteristics (Supplementary Table 3).

Table 2 shows the external validation metrics. In L&H-risk individuals, the specificity was similar in both sexes (men: 0.795; women 0.774, *i*-value = 0.783). The NPV was extremely high (men: 0.898; women: 0.941). Thus, PASAP-ILERVAS showed a high ability in identifying truly healthy individuals.

The PPV was higher in men than in women (0.561 vs. 0.178, *p*-value: < 0.001). It is noteworthy that performance metrics were limited by the low prevalence of SA in the external validation cohort (men: 31.2%; women: 10.7%). Sensitivity was similar in both sexes (0.744 vs. 0.500, *p*-value = 0.141). Accuracy was high in both, indicating a 78.2% and a 75.0% of correct predictions by the PASAP-ILERVAS. Finally, the AUC was 0 .799 in men and 0.691 in women.

### Comparison of the Personalized Algorithm for Severe Atheromatosis Prediction Algorithm With the Systematic COronary Risk Evaluation

**FIGURE 5:**
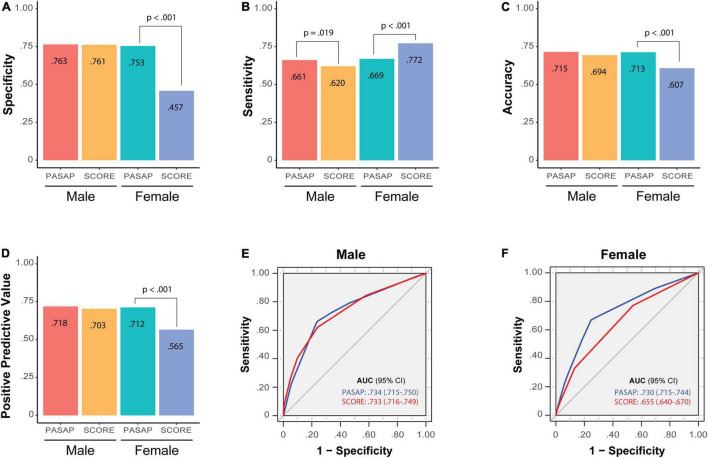
Comparison of the PASAP-ILERVAS with the SCORE risk score. Barplots contain the main performance metrics of PASAP-ILERVAS and SCORE risk model in L&H-risk individuals: **(A)** specificity; **(B)** sensitivity; **(C)** accuracy; **(D)** positive predictive model; **(E)** ROC curve in men; **(F)** ROC curve in women. The ROC curves reflect the area under the curve between sensitivity and complementary of specificity with both models. SCORE, Systematic COronary risk Evaluation; ROC, Receiver Operating Characteristic.

The performance of the PASAP-ILERVAS to predict SA in L&H-risk individuals was compared to the traditional SCORE model (Figure 5 and Table 3).

**TABLE 3:** Improvement in risk prediction of PASAP-ILERVAS respect to SCORE model.

	Men	Women
**NRI**	0.044 (0.020, 0.068)	0.193 (0.162, 0.224)
**IDI**	0.038 (0.029, 0.048)	0.119 (0.109, 0.129)
**ΔAUC**	0.001 (–0.012, 0.013)	0.074 (0.062, 0.087)

*The increment on AUC (ΔAUC), the Net Reclassification Index (NRI), and the Integrated Discrimination Index (IDI) with 95% CI were shown.*

In men, the PASAP-ILERVAS showed a higher sensitivity than the traditional score (0.661 vs. 0.620, *p*-value: 0.019). Thus, the new algorithm had a higher ability to detect L&H-risk men who actually were affected by the disease. In contrast, other metrics were very similar in both models (specificity: 0.763 vs. 0.761, *p*-value: 0.903; PPV: 0.718 vs. 0.703, *p*-value: 0.398; and accuracy: 0.715 vs. 0.694, *p*-value: 0.070). However, although the increment of AUC was not significant (*p*-value: 0.962), the PASAP-ILERVAS showed an NRI of 0.044 (95% CI: 0.020–0.068) and an IDI of 0.038 (95% CI: 0.029–0.048) compared to the SCORE model.

In women, the PASAP-ILERVAS showed a higher specificity than the SCORE (0.753 vs. 0.457, *p*-value: < 0.001), a lower sensitivity (0.669 vs. 0.772, *p*-value: < 0.001), and a higher accuracy (0.713 vs. 0.607, *p*-value: < 0.001). The PPV was also higher in the PASAP-ILERVAS (0.712 vs. 0.565, *p*-value: < 0.001). The new algorithm showed a significant increase in the AUC of 0.074 (95% CI: 0.062–0.087, *p*-value: < 0.001). Thus, PASAP-ILERVAS showed a higher percentage of correct predictions in L&H-risk women than the traditional SCORE, especially those women who truly had severe atheromatosis, showing an NRI of 0.193 (95% CI: 0.162–0.224) and an IDI of 0.119 (95% CI: 0.109–0.129) compared to the SCORE model.

## Discussion

In this study, we presented a novel, sex-specific, machine learning-based algorithm for SA prediction in asymptomatic middle-aged individuals. The PASAP-ILERVAS integrates clinical history data, anthropometrical measurements, and affordable biochemical parameters to obtain a hierarchical, flexible, easy-to-interpret, predictive model. This model was validated in an external cohort, showing excellent performance in the L&H-risk individuals. Thus, the algorithm could be useful to select individuals who can benefit from a vascular ultrasound, namely those classified as intermediate risk. Non-invasive imaging techniques can detect the presence of atheromatosis and estimate the burden, which has been proven to improve cardiovascular risk assessment (7), and it is recommended when the coronary artery calcium score is unavailable or not feasible (41). Furthermore, it is a cheap and accessible in most medical offices. However, the implementation of vascular ultrasound, which could help increase accuracy in those patients has not been accomplished in the routine clinical practice due to several reasons. Among them, the high-time burden added to each visit is likely one. Therefore, identifying patients in whom a vascular ultrasound could add prognostic value is of paramount importance.

The novel PASAP-ILERVAS stratifies individuals according to their risk of SA as low, intermediate, and high. First, patients were classified by age, followed by smoking habit. The combination of these two parameters conditioned the clinical threshold of the other predictors. Thus, the ML approach evidenced the additive effect of cardiovascular risk factors. In addition, the algorithm revealed that biological thresholds which conditioned ASCVD risk, must be considered individually.

Age, smoking habit, SBP, and total cholesterol accounts 98.4% of the predictor importance in men and 82.3% in women. Additionally, a GFR ≥ 107 ml/min/m^2^ increased the risk in men. Although it is amply proven that kidney failure increases ASCVD risk as kidney function decreases, few studies linked an increased glomerular filtration with ASCVD risk. Glomerular hyperfiltration is an initial step of kidney damage in diabetes and obesity, but its threshold remains elusive. However, there are data showing that glomerular hyperfiltration of 107–115 ml/min/m^2^ is independently associated with increased cardiovascular risk in middle-aged healthy individuals (42). In women, other predictors related to obesity were identified. Obesity is associated with an increased cardiovascular risk since the pro-inflammatory cytokines produced by the adipose tissue itself induce atherosclerotic plaque formation (43), and a larger waist perimeter was independently associated with recurrent atherosclerotic cardiovascular disease (44). The PESA study, which is another large Spanish cohort similar to the ILERVAS cohort with asymptomatic middle-aged individuals without established CVD, revealed that in metabolically healthy individuals the presence of subclinical atherosclerosis increased across BMI categories, whereas fewer differences were observed for metabolically unhealthy individuals. Thus, the presence of subclinical atherosclerosis observed in patients with an abnormal BMI was mainly attributed to the coexistence of other cardiovascular risk factors, such as hypertension, diabetes, and dyslipidemia (45). A recent study in a subpopulation of the PESA study revealed that individuals with metabolic syndrome or its individual components (central obesity, hypertension, low HDL-C, triglycerides, and altered glucose metabolism) showed bone marrow activation, even in the absence of systemic inflammation, which is associated with early atherosclerosis (46). Similarly, uric acid levels have been positively correlated with cardiovascular diseases, including hypertension and atherosclerosis, through oxidative stress and an inflammatory response (47). Women are often older than men when they suffer from their first cardiovascular event (48). This difference is attributed to the protective role of circulating estrogens on the vascular endothelium, maintaining oxide nitric release leading to vasodilation (49), regulating prostaglandin production and inhibiting smooth muscle cell proliferation (50). On the contrary, at menopause, women show endothelial dysfunction and lipid deposition in the artery walls, which can promote atherosclerosis development (51). In women under 55 years of age, smoking is the most important preventable cause of CVD, increasing their risk 7-fold (52). Large cohort studies have demonstrated that high SBP is an important risk factor for cardiovascular disease and a 5-mm Hg reduction of SBP reduced the risk of major cardiovascular events by about 10% (53). Strikingly, hypertension is more strongly associated with CVD in women compared to men (54).

The PASAP-ILERVAS can be used as a prescreening model to determine patients who will further benefit from a vascular ultrasound examination. It identified three risk profiles (low, intermediate, and high). The internal validation showed that specificity, PPV, accuracy, and AUC were higher in L&H-risk individuals compared to the total sample (*p*-value: < 0.001), which indicate a higher feasibility of the PASAP-ILERVAS in those patients. In L&H-risk individuals, all parameters were similar in both sexes. The NPV and the PPV were higher than 71%, which indicated that PASAP-ILERVAS algorithm correctly identified patients with SA (true positives) or without SA (true negatives). The external validation results were limited by the low prevalence of SA in that cohort. However, the accuracy was high in both sexes (men: 0.782; women: 0.750), indicating a high percentage of correct predictions. Thus, patients with an estimated low- or high-risk are very accurately classified by the algorithm and are not candidates for vascular ultrasound examination. On the contrary, patients in whom the prediction is not so accurate (intermediate-risk) are strong candidates to undergo a vascular ultrasound to truly determine whether they have SA in order to adjust treatments and/or follow-up visits. The NPV was extremely high (men: 0.898; women: 0.941). Thus, PASAP-ILERVAS showed a high ability in identifying truly healthy individuals.

The SCORE is a cardiovascular risk assessment tool that estimates the 10-year risk of fatal CVD (55). At the moment, there is no internationally accepted tool to predict subclinical atheromatosis, so new methods to do so have been previously compared to the SCORE in similar middle-aged asymptomatic cohorts (18, 56, 57). The comparison of the PASAP-ILERVAS with the SCORE model revealed a better performance in both sexes, showing a higher sensitivity in men, and a higher PPV, specificity, accuracy, and AUC in women. Importantly, PASAP-ILERVAS improved patients’ reclassification in both sexes, but was much higher in women [NRI in men: 0.044 (95% CI: 0.020–0.068); women: 0.193 (95% CI: 0.162–0.224)]. Thus, the new algorithm showed a higher percentage of correct predictions in L&H-risk women than the traditional SCORE, especially in healthy women (specificity:0.753 vs. 0.457, *p*-value: < 0.001) and in those who were positive in screening test and truly had severe atheromatosis (PPV:0.712 vs. 0.565, *p*-value: < 0.001). Indeed, our algorithm showed a significant increase in the AUC of 0.074 (95% CI: 0.062–0.087, *p*-value: < 0.001) in women.

Several limitations should be considered when interpreting this study. First, results are based on a cross-sectional analysis from the ILERVAS study cohort. However, the inclusion of history factors, such as hypertension and dyslipidemia, partially overcomes this limitation. Second, the ILERVAS cohort consists of middle-aged symptomatic participants with relatively homogeneous socioeconomic, lifestyle, and ethnic characteristics. However, having a homogenous population increases internal validity, can unveil hidden clinical associations, and is not uncommon (45, 58). Even though the PASAP-ILERVAS was externally validated in another cohort, further analysis would be considered to reinforce data extrapolation. Third, the derivation cohort is different not only in size (< 10% of individuals), and time period, but also in terms of incidence of subclinical atheromatosis, and even baseline characteristics of the patients. Fourth, the levels of glycosylated hemoglobin can be used to identify asymptomatic individuals at higher risk of subclinical atherosclerosis on top of traditional cardiovascular risk factors (58). However, unfortunately, we could not study the contribution of HbA1c in the PASAP-ILERVAS due to a high percentage of missing values in the external validation cohort. Finally, recursive partitioning may be complex or overfit the data. This issue was addressed by a post-pruning technique based on the CP, which shows the trade-off between the tree complexity and how well the tree fits the data.

In contrast, our study has several strengths. First, the study population was randomized, and a stratified sampling was performed from primary care records to reduce selection bias, and to obtain a representative cohort of the entire province. Second, contrary to recent atheromatosis prediction algorithm, a sex-stratified analysis was performed to unveil clinical differences in men and women. Third, rpart is a non-parametric ML method that can handle highly skewed data, does not require data categorization, and generates an easy-to-interpret graphical representation, which is very convenient in the daily clinical practice. Finally, the algorithm combines clinical history data, anthropometrical measurements, and affordable biochemical parameters that can be easily obtained in non-hospital settings, such as primary care centers and even pharmacies. Blood biochemical parameters were obtained by dried blood spot tests, which are highly validated methods.

## Conclusion

We developed a personalized, sex-specific, easy-to-interpret model for severe atheromatosis prediction in asymptomatic middle-aged individuals. The PASAP-ILERVAS predicted accurately in L&H-risk patients. However, in intermediate-risk individuals a vascular imaging exploration would be recommended. Thus, the present algorithm could reduce the number of unnecessary complementary explorations selecting candidates for a further imaging study, increasing cost-effectiveness and optimizing health resources.

## Data Availability Statement

The raw data supporting the conclusions of this article will be made available by the authors, without undue reservation.

## Ethics Statement

The studies involving human participants were reviewed and approved by CEIC Hospital Universitario Arnau de Vilanova. The patients/participants provided their written informed consent to participate in this study.

## Ilervas Project Collaborators

Eva Miquel and Marta Ortega: Centre d’Atenció Primària Cappont, Gerència Territorial de Lleida, Institut Català de la Salut, Barcelona, Spain; Research Support Unit Lleida, Fundació Institut Universitari per a la recerca a l’Atenció Primària de Salut Jordi Gol i Gorina (IDIAPJGol), Barcelona, Spain; Ferran Barbé, Jessica González, Silvia Barril, and Manuel Sánchez-de-la-Torre: Departament de Medicina Respiratòria, Hospital Universitari Arnau de Vilanova, Grup Recerca Translational Medicina Respiratòria, IRBLleida, Universitat de Lleida, Lleida, Spain; CIBER de enfermedades respiratorias (CIBERES), Madrid, Spain; Manuel Portero-Otín and Mariona Jové: Departament de Medicina Experimental, IRBLleida, Universitat de Lleida, Lleida, Spain; Marta Hernández and Ferran Rius: Departament d’Endocrinologia i Nutrició, Hospital Universitari Arnau de Vilanova, Grup de Recerca Obesitat i Metabolisme (ODIM), IRBLleida, Universitat de Lleida, Lleida, Spain; Centro de Investigación Biomédica en Red de Diabetes y Enfermedades Metabólicas Asociadas (CIBERDEM), Instituto de Salud Carlos III (ISCIII), Madrid, Spain; Josep Franch-Nadal and Esmeralda Castelblanc: Centro de Investigación Biomédica en Red de Diabetes y Enfermedades Metabólicas Asociadas (CIBERDEM), Instituto de Salud Carlos III (ISCIII), Madrid, Spain; Departament d’Endocrinologia i Nutrició, Hospital de la Santa Creu i Sant Pau, Institut de Recerca Biomèdica Sant Pau (IIB Sant Pau), Barcelona, Spain; Pere Godoy: Agència de Salut Pública de Catalunya, Departament de Salut, IRBLleida, Universitat de Lleida, Lleida, Spain; CIBER de Epidemiología y Salud Pública (CIBERESP), Madrid, Spain; Montse Martinez-Alonso: Unitat de Bioestadística, IRBLleida, Departament de ciències Mèdiques Bàsiques, Universitat de Lleida, Lleida, Spain.

## Author Contributions

MB-L, JV, and EF: study concept and design. EC-B and CF: data acquisition. MB-L, MM-A, JV, EF, and MB: data interpretation. MM-A and MB-L: statistical analysis and drafting of the manuscript. EF: study supervision. ILERVAS investigators: collected baseline and prospective follow-up data of the cohort. All authors contributed to the critical revision of the manuscript for important intellectual content and article and approved the submitted version.

## Conflict of Interest

The authors declare that the research was conducted in the absence of any commercial or financial relationships that could be construed as a potential conflict of interest.

## Publisher’s Note

All claims expressed in this article are solely those of the authors and do not necessarily represent those of their affiliated organizations, or those of the publisher, the editors and the reviewers. Any product that may be evaluated in this article, or claim that may be made by its manufacturer, is not guaranteed or endorsed by the publisher.
