# Supplementary material for: Development and Validation of a Personalized, Sex-Specific Prediction Algorithm of Severe Atheromatosis in Middle-Aged Asymptomatic Individuals: The ILERVAS Study
Source: Front Cardiovasc Med. 2022 Jul 14;9:895917. doi: 10.3389/fcvm.2022.895917 (PMC9344070; doi:10.3389/fcvm.2022.895917)
Supplement: Supplementary file 1 [file Data_Sheet_1.pdf]

SUPPLEMENTARY MATERIAL

1. SUPPLEMENTARY RESUTS

Table S1. Missing values of the ILERVAS and NEFRONA cohorts.

	**ILERVAS COHORT** (Discovery) n=14		**NEFRONA COHORT** (Validation) n=399	
	**Severe atheromatosis**		**Severe atheromatosis**	
	**No**	**Yes**	**No**	**Yes**
**Anthropometrical data**				
Body mass index	1	1	0	0
Abdominal adiposity	1	2	0	0
Waist-to-height ratio	1	3	0	0
**Biochemical parameters**				
Creatinine	3	0	0	0
Glycosylated hemoglobin	1	0	238	77
Uric acid	1	0	45	18
Total cholesterol	0	0	18	3

Table S2. Association of age-adjusted clinical parameters with severe atheromatosis in the ILERVAS cohort.

	Males			Females		
	OR	95% CI	p-value	OR	95% CI	p-value
Age	1.76	1.65-1.88	<.001	1.51	1.41-1.63	<.001
SBP	1.32	1.24-1.42	<.001	1.23	1.15-1.33	<.001
DBP	1.17	1.10-1.25	<.001	1.07	1.00-1.15	.060
Creatinine	0.86	0.80-0.93	<.001	0.95	0.89-1.02	.181
GFR	0.95	0.89-1.01	.094	0.91	0.85-0.97	.007
Glycosylated hemoglobin	1.11	1.04-1.18	.002	1.09	1.02-1.17	.014
Uric acid	1.10	1.03-1.17	.003	1.10	1.03-1.18	.007
Total Cholesterol	1.24	1.16-1.32	<.001	1.13	1.05-1.21	<.001
Smoking habit						
Non-smoker vs rest	0.34	0.28-0.40	<.001	0.42	0.36-0.50	<.001
Former vs non-smoker	2.22	1.85-2.66	<.001	1.70	1.38-2.08	<.001
Current vs non-smoker	4.33	3.55-5.21	<.001	3.46	2.82-4.25	<.001
Current vs former	2.51	2.18-2.89	<.001	2.89	2.42-3.45	<.001
BMI						
Underweight vs normal	7.02	1.43-34.50	.006	3.39	1.41-8.17	.008
Overweight/obesity vs underweight	0.14	0.03-0.65	.004	0.27	0.12-0.65	.004
Abdominal adiposity	1.03	0.90-1.17	.688	0.98	0.80-1.21	.847
Clinical history						
Hypertension	1.24	1.09-1.42	.002	1.58	1.36-1.83	<.001
Dyslipidemia	1.19	1.05-1.36	.007	1.21	1.04-1.40	.012

Univariate logistic regressions adjusted by age. Odds Ratio (OR) values and 95% confidence intervals (95% CI) were shown. Clinical history data were obtained from electronic medical records and refers to patients who had prior clinical diagnostic of hypertension or dyslipidemia. Underweight

was defined as a BMI < 18.5 kg/m^2, normal weight as 18.5-24.9, overweight 25-29.9, and obesity ≥ 30. Abdominal adiposity was defined as an abdominal perimeter ≥ 88 cm in women or ≥ 102 in men. For each numerical predictor, adjusted OR per 1-SD higher parameter measure was estimated. SD for males and females were: SBP: 16.07-17.25 mmHg, DBP: 9.42-9.00 mmHg, creatinine: 0.20-0.16 mg/dL, GFR: 14.20-14.14 mL/min/1.73 m^2; Glycosylated hemoglobin: 0.45-0.38%; total cholesterol: 37.85-36.67 mg/dL; Uric acid: 1.40-1.29 mg/dL. BMI: body mass index; DBP: diastolic blood pressure; GFR: glomerular filtration rate; SBP: systolic blood pressure.

Table S3. Baseline characteristics of the NEFRONA cohort by sex.

	**Males** n=298			**Females** n=261		
	**Severe atheromatosis**			**Severe atheromatosis**		
	**No**	**Yes**	*p-Value*	**No**	**Yes**	*p-Value*
n (%)	203 (68.1%)	95 (31.9%)		230 (88.1%)	31 (11.9%)	
Age, years	52.57 (11.53)	61.92 (6.82)	<.001	52.46 (12.12)	61.48 (7.38)	<.001
**Clinical history**, n (%)						
Hypertension	118 (58.1%)	82 (86.3%)	.010	83 (36.1%)	23 (74.2%)	.012
Dyslipidemia	69 (34.0%)	46 (48.4%)	.145	63 (27.4%)	19 (61.3%)	.014
Smoking habit			.002			.816
Non-smoker	76 (37.4%)	15 (15.8%)		113 (49.1%)	18 (58.1%)	
Former	86 (42.4%)	58 (61.1%)		75 (32.6%)	9 (29.0%)	
Current	41 (20.2%)	22 (23.2%)		42 (18.3%)	4 (12.9%)	
**Anthropometrical data**						
SBP, mmHg	135.44 (16.09)	142.64 (16.11)	.183	126.42 (16.69)	140.65 (20.13)	.010
DBP, mmHg	81.10 (8.99)	83.41 (8.37)	.040	77.66 (10.28)	79.90 (9.91)	.572
Body mass index, n (%)			.199			.527
Underweight	0 (0.0%)	0 (0.0%)		4 (1.7%)	0 (0.0%)	
Normal weight	39 (19.2%)	8 (8.4%)		77 (33.5%)	10 (32.3%)	
Overweight/Obesity	164 (80.8%)	87 (91.6%)		149 (64.8%)	21 (67.7%)	
Abdominal adiposity, n (%)	77 (37.9%)	44 (46.3%)	.680	153 (66.5%)	22 (71%)	.711
Waist-to-height ratio	0.58 (0.06)	0.61 (0.06)	.219	0.58 (0.08)	0.61 (0.08)	.563
**Biochemical parameters**						

Creatinine, mg/dL	0.92 (0.14)	0.94 (0.12)	.346	0.74 (0.11)	0.72 (0.10)	.198
GFR, mL/min/1.73 m^2	92.74 (14.49)	85.46 (10.70)	<.001	92.77 (14.21)	88.56 (12.17)	.114
Glycosylated hemoglobin, %	5.52 (0.78)	6.47 (1.38)	.009	5.42 (1.02)	5.96 (0.76)	.500
Uric acid, mg/dL	5.79 (1.22)	5.96 (1.20)	.307	4.12 (1.10)	4.68(1.11)	.077
Total cholesterol, mg/dL	197.77 (31.54)	206.00 (36.57)	.037	205.94 (36.67)	213.39 (46.19)	.590

Values are shown as means and standard deviations for quantitative variables. Clinical history data were obtained from electronic medical records and refers to patients who had prior clinical diagnostic of hypertension or dyslipidemia. Underweight was defined as a BMI < 18.5 kg/m^2, normal weight as 18.5-24.9, overweight 25-29.9, and obesity ≥ 30. Abdominal adiposity was defined as an abdominal perimeter ≥ 88 cm in women or ≥ 102 in men. DBP: diastolic blood pressure; GFR: glomerular filtration rate; SBP: systolic blood pressure.

2. SUPPLEMENTAL FIGURE LEGENDS

Figure S1. Association of age-adjusted clinical parameters with severe atheromatosis in the ILERVAS cohort.

Univariate logistic regressions adjusted by age. Odds Ratio (OR) values and 95% confidence intervals (95% CI) were represented. Clinical history data were obtained from electronic medical records and refers to patients who had prior clinical diagnostic of hypertension or dyslipidemia. Underweight was defined as a BMI < 18.5 kg/m^2, normal weight as 18.5-24.9, overweight 25-29.9, and obesity ≥ 30. Abdominal adiposity was defined as an abdominal perimeter ≥ 88 cm in women or ≥ 102 in men. For each numerical predictor, adjusted OR per 1-SD higher parameter measure was estimated. SD for males and females were: SBP: 16.07-17.25 mmHg, DBP: 9.42-9.00 mmHg, creatinine 0.20-0.16 mg/dL, GFR: 14.20-14.14 mL/min/1.73 m^2; Glycosylated hemoglobin: 0.45-0.38%; total cholesterol: 37.85-36.67 mg/dL; Uric acid: 1.40-1.29 mg/dL. BMI: body mass index; DBP: diastolic blood pressure; GFR: glomerular filtration rate; SBP: systolic blood pressure.

Figure S2. Graphical output of the PASAP-ILERVAS.

The relationship between complexity parameter (downside x-axis), cross-validation error (y-axis) and number of splits (upside x-axis) were represented. The horizontal dotted line represented the cut-off cross-validation error statistic at one SD above minimal cross-validation-error. The three-fold cross-validation suggested pruning the tree back to 13 splits in males and 17 splits in females showed the lowest cross-validation error, beyond which tree complexity entailed no additional improvement.

Figure S3. Histogram-transformed calibration of PASAP-ILERVAS probabilities.

The process of PASAP-ILERVAS probability calibration was represented. First, the original probabilities with a maximum of 10 bins were plotted (pre-calibration). Then, the optimal number of partitions to maximize the sensitivity was tested. The calibration process evidenced three risk groups (post-calibration), classified as low-, intermediate-, and high-risk nodes. The absolute frequencies of patients with and without the disease inside the bins were coloured in green or red over the Y-axis, respectively. The probabilities of severe atheromatosis were represented in the X-axis.
