# Supplementary material for: Development and Validation of a Personalized, Sex-Specific Prediction Algorithm of Severe Atheromatosis in Middle-Aged Asymptomatic Individuals: The ILERVAS Study
Source: Front Cardiovasc Med. 2022 Jul 14;9:895917. doi: 10.3389/fcvm.2022.895917 (PMC9344070; doi:10.3389/fcvm.2022.895917)

Figure S1

Association of clinical parameters with severe atheromatosis in the ILERVAS cohort

a)

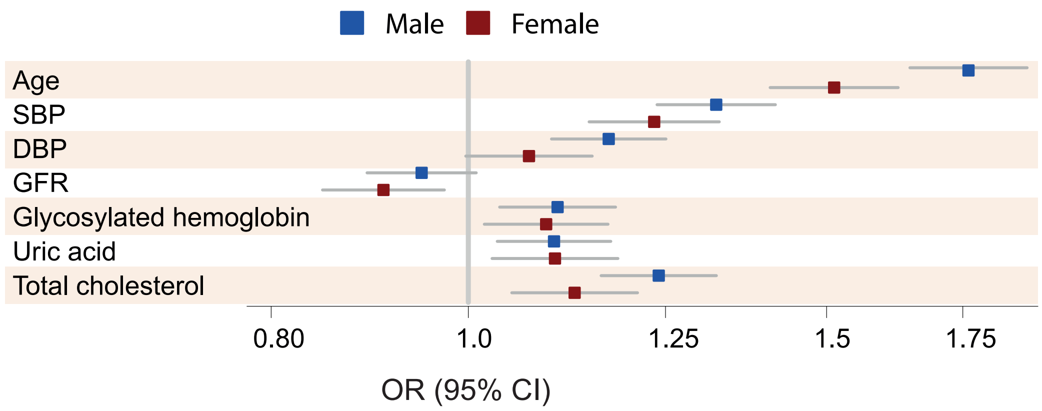

b)

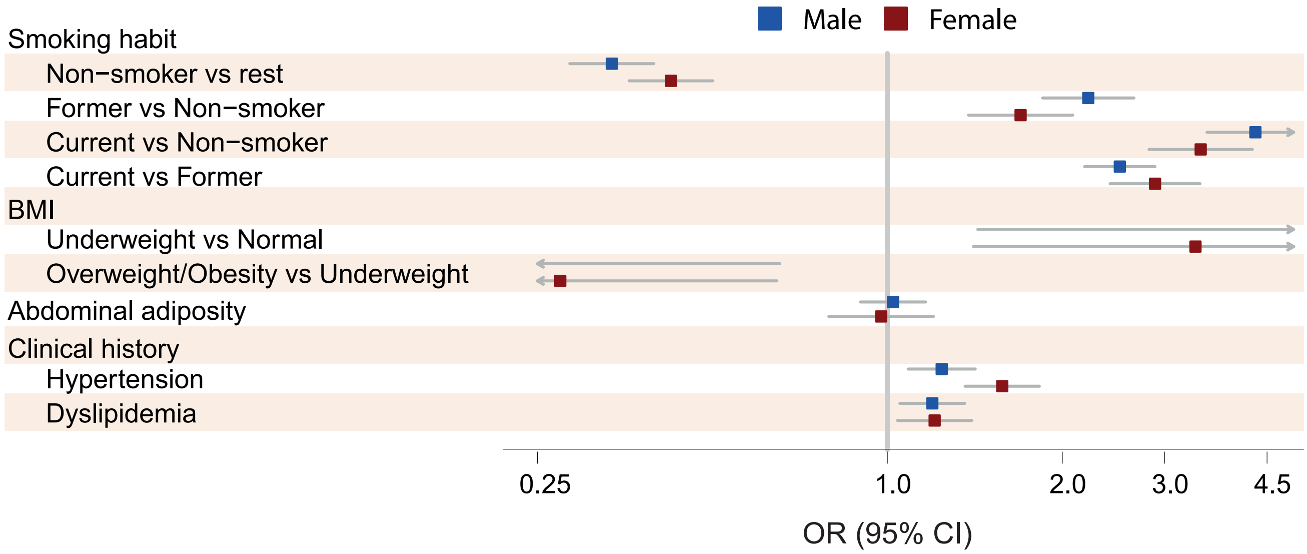

Supplement: Supplementary file 2 [file Data_Sheet_2.PDF]
